# Venous Thromboembolism After COVID-19 Infection Among People With and Without Immune-Mediated Inflammatory Diseases

**DOI:** 10.1001/jamanetworkopen.2023.37020

**Published:** 2023-10-09

**Authors:** Rabia Khan, M. Ellen Kuenzig, Furong Tang, James H. B. Im, Jessica Widdifield, Jeffrey D. McCurdy, Gilaad G. Kaplan, Eric I. Benchimol

**Affiliations:** 1 SickKids Inflammatory Bowel Disease Centre, Division of Gastroenterology, Hepatology and Nutrition, The Hospital for Sick Children, Toronto, Ontario, Canada; 2 Child Health Evaluative Sciences, SickKids Research Institute, The Hospital for Sick Children, Toronto, Ontario, Canada; 3 ICES, Toronto, Ontario, Canada; 4 Sunnybrook Research Institute, Toronto, Ontario, Canada; 5 Institute of Health Policy, Management and Evaluation, University of Toronto, Toronto, Ontario, Canada; 6 Department of Medicine, University of Ottawa, Ottawa, Ontario, Canada; 7 Ottawa Hospital Research Institute, Ottawa, Ontario, Canada; 8 Division of Gastroenterology, The Ottawa Hospital IBD Centre, Ottawa, Ontario, Canada; 9 Departments of Medicine and Community Health Sciences, University of Calgary, Calgary, Alberta, Canada; 10 Department of Paediatrics, University of Toronto, Toronto, Ontario, Canada

## Abstract

**Importance:**

Immune-mediated inflammatory diseases (IMIDs) and COVID-19 are independently associated with venous thromboembolisms (VTEs).

**Objective:**

To determine if individuals with IMIDs are at higher risk of VTE following COVID-19 infection compared with individuals without IMIDs.

**Design, Setting, and Participants:**

Population-based matched cohort study using multiple deterministically linked health administrative databases from Ontario, Canada, and including patients testing positive for COVID-19 between January 1, 2020, and December 30, 2021, and followed up until March 31, 2022. Individuals with IMIDs (n = 28 440) who tested positive for COVID-19 were matched with up to 5 individuals without an IMID (n = 126 437) who tested positive for COVID-19. Matching was based on year of birth, sex, neighborhood income, and rural/urban residence. Data analysis was performed from August 6, 2022, to August 21, 2023.

**Exposure:**

Diagnosis of an IMID, identified using algorithms based on diagnostic codes, procedures, and specialist visits.

**Main Outcome and Measure:**

The main outcome was estimated age- and sex-standardized incidence of VTE. Proportional cause-specific hazard models compared the risk of VTE in people with and without IMIDs. Death was a competing risk. Models adjusted for history of VTE, 2 or more doses of a COVID-19 vaccine 14 or more days prior to COVID-19 diagnosis, and the Charlson Comorbidity Index. Routinely collected health data were used, so the hypothesis tested was formulated after data collection but prior to being granted access to data.

**Results:**

The study included 28 440 individuals (16 741 [58.9%] female; 11 699 [41.1%] male) with an IMID diagnosed prior to first COVID-19 diagnosis, with a mean (SD) age of 52.1 (18.8) years at COVID-19 diagnosis. These individuals were matched to 126 437 controls without IMIDs. The incidence of VTE within 6 months of COVID-19 diagnosis among 28 440 individuals with an IMID was 2.64 (95% CI, 2.23-3.10) per 100 000 person-days compared with 2.18 (95% CI, 1.99-2.38) per 100 000 person-days among 126 437 matched individuals without IMIDs. The VTE risk was not statistically significantly different among those with vs without IMIDs (adjusted hazard ratio, 1.12; 95% CI, 0.95-1.32).

**Conclusions and Relevance:**

In this retrospective population-based cohort study of individuals with IMIDs following COVID-19, individuals with IMIDs did not have a higher risk of VTE compared with individuals without an IMID. These data provide reassurance to clinicians caring for individuals with IMIDs and COVID-19.

## Key Points

**Question:**

Are individuals with immune-mediated inflammatory diseases (IMIDs) more likely to experience a venous thromboembolism (VTE) following COVID-19 infection than individuals without IMIDs?

**Findings:**

In a matched cohort study conducted using health administrative data from Ontario, Canada, the incidence of VTE among 28 440 individuals with an IMID was 2.64 per 100 000 person-days compared with 2.18 per 100 000 person-days among 126 437 individuals without an IMID. After adjusting for having 2 or more doses of a COVID-19 vaccine, history of VTE, and comorbidities, the risk of VTE was not statistically significantly different for people with IMIDs.

**Meaning:**

Following a COVID-19 diagnosis, the overall risk of VTE is low among individuals with IMIDs.

## Teaser

This cohort study assesses risk of venous thromboembolism following COVID-19 infection in individuals with vs without immune-mediated inflammatory diseases in Ontario, Canada.

## Introduction

COVID-19 may cause widespread inflammation; hyperactivation of this systemic immune response may ultimately cause multiorgan failure and death.^1,2,3^ Venous thromboembolisms (VTEs) are known complications following COVID-19^4,5,6^ and are associated with significant morbidity and mortality.

Immune-mediated inflammatory diseases (IMIDs) are a heterogeneous group of chronic diseases that result from abnormal activation of the immune system, affecting 5% to 7% of people in the Western world.^7^ Individuals with IMIDs are at a higher risk of VTE than the general population, particularly during disease flares,^8,9^ potentially due to inflammation causing endothelial dysfunction, platelet abnormalities, activation of the coagulation system, and impaired fibrinolysis. Inflammatory bowel disease (IBD),^10,11,12,13^ rheumatoid arthritis,^14,15^ psoriasis,^16^ vasculitis,^17^ and multiple sclerosis^18^ have all been reported to be associated with higher VTE risk.

The interplay between COVID-19, IMIDs, and VTE remains undefined. We hypothesized that individuals with IMIDs may have an elevated risk of VTE following COVID-19. We aimed to evaluate the incidence and risk of VTE following COVID-19 in individuals with IMIDs compared with those without IMIDs using population-based health administrative data from Ontario, Canada.

## Methods

### Data Sources

This study used health administrative data from Ontario, Canada, which has a publicly funded universal health care system. Data include all health care interactions of Ontario residents with valid health cards (>99% of the population). This includes hospitalizations (Canadian Institute for Health Information [CIHI] Discharge Abstract Database), procedures requiring single-day hospital admission (CIHI Same Day Surgery Database), emergency department visits (CIHI National Ambulatory Care Reporting System), and visits to the hospital for outpatient surgeries or other procedures (CIHI Same Day Surgery Database). Data also include physician billings for all patient-physician interactions, including outpatient visits; these data include physician specialty. Data were linked deterministically to the Registered Persons Database (demographic characteristics), Ontario Cancer Registry, C19INTGR (COVID-19 testing), and COVaxON (COVID-19 vaccinations). All data are held by ICES, an independent, nonprofit research institute whose legal status under Ontario’s health information privacy law allows it to collect and analyze health care and demographic data, without consent, for health system evaluation and improvement. Data are available in uncleaned, unedited format to researchers and data analysts. The use of the data in this project is authorized under section 45 of Ontario’s Personal Health Information Protection Act (PHIPA) and does not require review by a research ethics board. This study was reported in accordance with the Reporting of Studies Conducted Using Observational Routinely Collected Health Data (RECORD), an extension to the Strengthening the Reporting of Observational Studies in Epidemiology (STROBE) reporting guideline for research using routinely collected health administrative data.^19^

### Study Design and Eligibility

We conducted a retrospective matched cohort study. Individuals with an IMID who tested positive for COVID-19 were randomly matched with up to 5 individuals without any IMIDs who tested positive for COVID-19 based on year of birth (±1 year), sex, mean neighborhood income quintile, and rural/urban residence. Before matching, we excluded individuals without a valid Ontario health card number, date of birth, date of death (date of death preceded date of positive COVID-19 test result), or sex. We also excluded people diagnosed with a malignant neoplasm within 5 years prior to testing positive for COVID-19.

### Identifying Individuals Testing Positive for COVID-19

Individuals testing positive for COVID-19 were identified from C19INTGR, which includes the results of all polymerase chain reaction (PCR) tests conducted in Ontario from January 1, 2020, to December 30, 2021, after which time PCR testing was limited to selected groups. Participants were followed up from the date of the first positive COVID-19 test result until VTE, death, migration out of Ontario, or end of follow-up (March 31, 2022).

### Identifying Individuals With IMIDs

Individuals with at least 1 diagnosis of ankylosing spondylitis, IBD, multiple sclerosis, polymyalgia rheumatica, psoriasis, psoriatic arthritis, rheumatoid arthritis, systemic autoimmune rheumatic disease (encompassing connective tissues disorders, such as lupus and scleroderma), uveitis, or vasculitis were identified using algorithms based on multiple health care encounters with disease-specific diagnostic codes (for hospitalizations, *International Classification of Diseases, Ninth Revision [ICD-9]* before April 1, 2002, and *International Statistical Classification of Diseases and Related Health Problems, Tenth Revision [ICD-10]* on or after April 1, 2002), the specialty of the physician submitting the billing claim for physician claims, procedure codes for endoscopy (IBD only), and indication-specific medications. Validated algorithms were available for IBD,^20,21^ multiple sclerosis,^22^ psoriasis,^23^ psoriatic arthritis,^23^ and rheumatoid arthritis.^24,25^ See eTable 1 in Supplement 1 for a description of the algorithms used to identify each of the IMIDs. We looked back until 1991 (the start of Ontario’s health administrative data) for incident or prevalent IMID diagnoses, except for vasculitis (April 1, 2002, due to improvements with *ICD-10* coding system introduction). Only individuals for whom the first diagnostic code for an IMID occurred before the date of COVID-19 diagnosis were included.

### Identifying Individuals With VTE

Venous thromboembolisms, including pulmonary embolism (PE) and deep vein thrombosis (DVT), were identified from emergency department and hospitalization data using *ICD-10* codes (eTable 2 in Supplement 1). Diagnostic codes used to identify VTE were previously validated in Alberta, Canada (sensitivity, 75.0%; specificity, 93.8%; positive predictive value, 73.3%; negative predictive value, 94.3%, when combined with radiology codes).^26^ Radiology codes used in the validation study were not widely used in patients with VTE in Ontario and were excluded from the identification algorithm. Our primary outcome was the first VTE of any type. Secondary outcomes were PE and DVT.

### Covariates and Matching Characteristics

#### Comorbidities

We used the Deyo adaptation of the Charlson Comorbidity Index (CCI), converted from *ICD-9* to *ICD-10* coding.^27,28,29,30^ We adapted the index to (1) include comorbidities treated in an outpatient setting and (2) remove codes used in our IMID-identification algorithms to avoid inflation of the CCI in people with IMIDs (see eTable 3 in Supplement 1 for list of excluded codes). We calculated CCI using data from the 5 years prior to COVID-19 diagnosis.

We report (1) the total number of IMIDs (among those with IMIDs; categorical: 1, 2, or ≥3); (2) the number of IMIDs in addition to the IMID of interest (among those with IMIDs; categorical: 0, 1, or ≥2); (3) IMID duration at the time of COVID-19 diagnosis; and (4) history of VTE in the 5 years prior to COVID-19 diagnosis. Patients with any recorded diagnosis of chronic obstructive pulmonary disease,^31^ congestive heart failure,^32^ or diabetes^33,34^ prior to their COVID-19 diagnosis were identified using previously validated algorithms (eTable 4 in Supplement 1).

Duration of IMID was calculated as the difference between the date of IMID diagnosis (date of the first code in the identification algorithm) and the date of COVID-19 diagnosis and categorized (<1, 1 to <3, 3 to <5, or ≥5 years). When the exact date of diagnosis was unknown (eg, a person did not meet a minimum lookback period), cases were assumed to be prevalent on the date of their first diagnostic code and assigned a disease duration of 5 or more years. When individuals had multiple IMIDs, IMID duration was defined as the time from the first IMID diagnosis to date of COVID-19 diagnosis.

#### Vaccination

COVaxON includes information on all COVID-19 vaccines administered in Ontario, Canada, and any out-of-province vaccines reported to public health. Anyone with 2 or more vaccinations 14 or more days before their positive COVID-19 test result was considered vaccinated.

#### Sociodemographic Characteristics

Sex, rural/urban residence, death prior to end of follow-up, and mean neighborhood income quintile (a validated proxy for individual-level socioeconomic status^35^) at the time of COVID-19 diagnosis were identified from the Registered Persons Database.

### Statistical Analysis

We summarized the characteristics of individuals with and without IMIDs using percentages for categorical variables. Continuous variables such as age were summarized using means with SDs. The CCI scores were summarized using medians with first and third quartiles.

The incidence of VTE (overall and stratified by type) was calculated by dividing the number of VTEs by the total person-time and reported as events per 100 000 person-days (PDs). We reported the crude and age- and sex-standardized incidence rates within 6 and 12 months of COVID-19 diagnosis using the 2017 Canadian population as the standard. Confidence intervals for both the crude and standardized incidence rates were calculated using the gamma method.^36^ Proportional cause-specific hazards models compared the risk of VTEs in people with and without IMIDs, treating death as a competing risk. We conducted a series of 3 models: (1) unadjusted, (2) adjusted for COVID-19 vaccination and history of VTE, and (3) adjusted for COVID-19 vaccination, history of VTE, and the CCI (continuous). We selected these variables as important confounders because of their impact on the magnitude of the association between IMIDs and VTE, with the number of events limiting the number of variables that could be included in a regression model.

Our primary analysis included individuals with any IMID. When a person had multiple IMIDs, we included them once in the analysis of all IMIDs and in disease-specific analyses. We conducted a sensitivity analysis censoring participants with a second COVID-19 diagnosis, applying a minimum look-forward period of 60 days to minimize the risk of censoring individuals following a second positive test result for the same infection. We also conducted a sensitivity analysis excluding patients with any history of VTE (after 2002).

All statistical tests were 2-sided with a 5% level of significance. Analyses were conducted using SAS, version 9.4 (SAS Institute Inc).

## Results

We identified 28 440 individuals with an IMID diagnosed prior to their first COVID-19 diagnosis, who were matched to 126 437 controls. Cases and controls were comparable for matched characteristics (Table 1). Patients with IMIDs had a mean (SD) age of 52.1 (18.8) years at COVID-19 diagnosis, and 58.9% were female, compared with the cohort without IMIDs, which had a mean (SD) age of 50.1 (18.0) years, and 58.4% were female. Psoriasis was the most common IMID (37.8%). The characteristics of included participants stratified by type of IMID are provided in eTable 5 in Supplement 1. We reported disease-specific analyses for all IMIDs except vasculitis, which was suppressed due to small cell sizes. Overall, we excluded 1332 individuals with IMIDs: 97 due to missing rural/urban status, and 1235 due to unsuccessful matching with any person without an IMID.

**Table 1.** Characteristics of Individuals With IMIDs and Matched People Without an IMID on the Date of Positive COVID-19 Test Result

Characteristic	No. (%)	
	People with any IMID (n = 28 440)	Matched people without an IMID (n = 126 437)
Age, mean (SD), y	52.1 (18.8)	50.1 (18.0)
Sex		
Female	16 741 (58.9)	73 793 (58.4)
Male	11 699 (41.1)	52 644 (41.6)
Rural	936 (3.3)	2014 (1.6)
Mean neighborhood income quintile		
1 (Lowest)	6095 (21.4)	27 179 (21.5)
2	5794 (20.4)	25 792 (20.4)
3	5876 (20.7)	26 410 (20.9)
4	5507 (19.4)	24 466 (19.4)
5 (Highest)	5168 (18.2)	22 590 (17.9)
Previous VTE	427 (1.5)	866 (0.7)
Chronic obstructive pulmonary disease	1286 (4.5)	3033 (2.4)
Congestive heart failure	1549 (5.4)	3907 (3.1)
Diabetes	5868 (20.6)	21 700 (17.2)
Charlson Comorbidity Index, median (IQR)	1 (0-2)	0 (0-1)
Type of IMID		
Ankylosing spondylitis	1453 (5.1)	NA
Inflammatory bowel disease	4331 (15.2)	NA
Multiple sclerosis	1954 (6.9)	NA
Psoriatic arthritis	1134 (4.0)	NA
Polymyalgia rheumatica	535 (1.9)	NA
Psoriasis	10 739 (37.8)	NA
Rheumatoid arthritis	5883 (20.7)	NA
Systemic autoimmune rheumatic disease	2506 (8.8)	NA
Uveitis	3000 (10.5)	NA
Vasculitis	512 (1.8)	NA
IMID duration		
<1 y	1115 (3.9)	NA
1 to <3 y	2762 (9.7)	NA
3 to <5 y	2695 (9.5)	NA
≥5 y	21 868 (76.9)	NA
No. of IMIDs		
1	25 479 (89.6)	NA
2	2411(8.5)	NA
≥3	550 (2.0)	NA
COVID-19 vaccination	7704 (27.1)	34 303 (27.1)
Deaths	15 661 (5.5)	4403 (3.5)

Abbreviations: IMID, immune-mediated inflammatory disease; NA, not applicable; VTE, venous thromboembolism.

**Figure 1.**
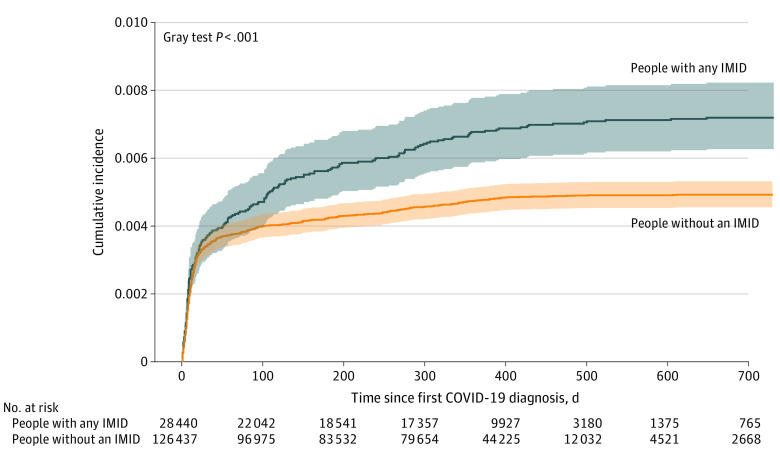
Cumulative Incidence of Venous Thromboembolism Among Individuals With and Without an Immune-Mediated Inflammatory Disease (IMID) Following COVID-19 Diagnosis

Within 6 months of COVID-19 diagnosis, the standardized incidence rate of VTE was 2.64 (95% CI, 2.23-3.10) per 100 000 PDs among people with IMIDs compared with 2.18 (95% CI, 1.99-2.38) among matched individuals without IMIDs (Figure 1; Table 2). Within 12 months of COVID-19 diagnosis, the standardized incidence rates were 1.82 (95% CI, 1.56-2.11) per 100 000 PDs among individuals with an IMID and 1.37 (95% CI, 1.25-1.49) among individuals without IMIDs. The highest standardized incidence of VTEs occurred among individuals with systemic autoimmune rheumatic disease (6.73 per 100 000 PDs; 95% CI, 3.13-12.62); the lowest, ulcerative colitis (0.79 per 100 000 PDs; 95% CI, 0.21-2.04).

**Table 2.** Incidence of Venous Thromboembolism Among Individuals With IMIDs and Matched Controls Within 6 Months and 12 Months of COVID-19 Diagnosis

IMID	People with an IMID		Matched people without an IMID	
	Crude IR per 100 000 PD (95% CI)	Standardized IR per 100 000 PD (95% CI)	Crude IR per 100 000 PD (95% CI)	Standardized IR per 100 000 PD (95% CI)
**Events within 6 mo of COVID-19 diagnosis**				
Any IMID	3.72 (3.17-4.35)	2.64 (2.23-3.10)	2.77 (2.54-3.02)	2.18 (1.99-2.38)
Ankylosing spondylitis	2.72 (1.00-5.93)	2.34 (0.81-5.27)	3.38 (2.34-4.72)	2.84 (1.88-4.10)
Inflammatory bowel disease	1.84 (0.95-3.21)	1.70 (0.78-3.21)	2.21 (1.71-2.81)	2.17 (1.58-2.91)
Crohn disease	2.86 (1.23-5.63)	3.54 (0.99-8.96)	1.94 (1.25-2.86)	2.69 (1.24-5.08)
Ulcerative colitis	1.16 (0.32-2.98)	0.79 (0.21-2.04)	2.55 (1.82-3.47)	2.20 (1.46-3.19)
Multiple sclerosis	3.03 (1.38-5.75)	3.03 (1.06-6.77)	2.14 (1.43-3.07)	1.77 (1.12-2.67)
Psoriasis	2.93 (2.16-3.88)	2.24 (1.63-3.00)	2.94 (2.57-3.36)	2.24 (1.94-2.58)
Psoriatic arthritis	4.06 (1.63-8.36)	5.71 (2.57-10.96)	2.19 (1.28-3.51)	1.29 (0.74-2.10)
Polymyalgia rheumatica	6.48 (2.10-15.12)	1.13 (0.26-3.14)	3.94 (1.97-7.06)	0.85 (0.36-1.68)
Rheumatoid arthritis	6.47 (4.91-8.36)	2.89 (0.91-6.85)	3.29 (2.75-3.92)	2.20 (1.76-2.71)
Systemic autoimmune rheumatic disease	6.06 (3.84-9.10)	6.73 (3.13-12.62)	2.07 (1.45-2.86)	1.75 (1.05-2.74)
Uveitis	4.20 (2.53-6.56)	2.91 (1.65-4.75)	2.97 (2.25-3.84)	1.78 (1.34-2.33)
**Events within 12 mo of COVID-19 diagnosis**				
Any IMID	2.60 (2.24-2.99)	1.82 (1.56-2.11)	1.79 (1.65-1.94)	1.37 (1.25-1.49)
Ankylosing spondylitis	2.14 (0.92-4.21)	1.79 (0.71-3.70)	2.03 (1.41-2.82)	1.66 (1.11-2.38)
Inflammatory bowel disease	1.56 (0.91-2.50)	1.48 (0.80-2.50)	1.47 (1.16-1.85)	1.43 (1.06-1.89)
Crohn disease	2.19 (1.05-4.02)	2.56 (0.82-6.02)	1.36 (0.91-1.96)	1.95 (0.87-3.74)
Ulcerative colitis	1.21 (0.48-2.48)	0.99 (0.38-2.10)	1.65 (1.20-2.22)	1.42 (0.96-2.02)
Multiple sclerosis	2.57 (1.37-4.39)	2.96 (1.22-6.00)	1.42 (0.98-1.99)	1.13 (0.74-1.66)
Psoriasis	2.17 (1.66-2.79)	1.61 (1.21-2.09)	1.91 (1.68-2.17)	1.41 (1.23-1.61)
Psoriatic arthritis	3.06 (1.40-5.81)	4.33 (1.47-9.84)	1.50 (0.92-2.32)	1.02 (0.59-1.64)
Polymyalgia rheumatica	5.07 (2.04-10.45)	4.29 (0.38-17.19)	2.38 (1.23-4.15)	0.53 (0.24-1.01)
Rheumatoid arthritis	4.06 (3.13-5.18)	2.02 (0.80-4.21)	2.09 (1.76-2.46)	1.37 (1.11-1.67)
Systemic autoimmune rheumatic disease	3.66 (2.35-5.45)	4.10 (1.82-7.92)	1.38 (1.00-1.87)	1.16 (0.73-1.75)
Uveitis	2.80 (1.75-4.23)	1.90 (1.12-3.01)	1.96 (1.52-2.48)	1.11 (0.85-1.43)

Abbreviations: IMID, immune-mediated inflammatory disease; IR, incidence rate; PD, person-days.

**Figure 2.**
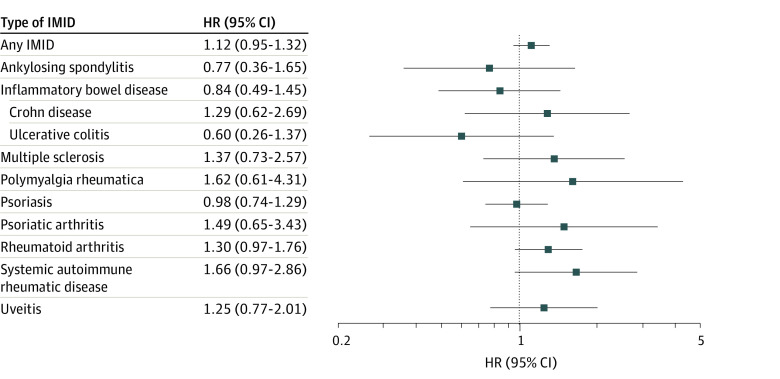
Forest Plot Depicting the Risk of Venous Thromboembolism Among Individuals With and Without (Reference) an IMID Following COVID-19 Diagnosis, Stratified by Type of IMID HR indicates hazard ratio; IMID, immune-mediated inflammatory disease.

In unadjusted analysis, people with IMIDs were significantly more likely to have a VTE compared with matched controls (hazard ratio [HR], 1.46; 95% CI, 1.24-1.71; eFigure 1 in Supplement 1). The association persisted when adjusted for previous VTE and COVID-19 vaccination (HR, 1.40; 95% CI, 1.20-1.64; eFigure 2 in Supplement 1). There was no significant association when additionally adjusting for comorbidities (adjusted HR [aHR], 1.12; 95% CI, 0.95-1.32; Figure 2). Results were consistent when stratifying by type of IMID and in sensitivity analysis censoring the 1584 individuals (1%) with a second COVID-19 diagnosis (aHR, 1.122 vs 1.121). In the sensitivity analysis excluding all people with a history of VTE after 2002 (n = 2566), there remained no statistically significant difference in risk of VTE in patients with IMIDs after adjusting for comorbidities and vaccination status (aHR, 1.07; 95% CI, 0.90-1.27).

Within 6 months of COVID-19 diagnosis, the standardized incidence rate of DVTs was 0.27 (95% CI, 0.14-0.46) per 100 000 PDs among individuals with IMIDs compared with 0.17 (95% CI, 0.12-0.23) among people without IMIDs (Table 3). Extending follow-up to 12 months, the standardized incidence rate of DVTs was 0.21 (95% CI, 0.12-0.33) per 100 000 PDs among individuals with IMIDs, compared with 0.12 (95% CI, 0.09-0.16) per 100 000 PDs among individuals without IMIDs. There was no difference in the risk of DVTs among those with and without an IMID (aHR, 0.99; 95% CI, 0.58-1.69).

**Table 3.** Incidence of Deep Vein Thrombosis and Pulmonary Embolism Among Individuals With IMIDs and Matched Controls Within 6 Months and 12 Months of COVID-19 Diagnosis

	People with an IMID		Matched people without an IMID	
	Crude IR per 100 000 PD (95% CI)	Standardized IR per 100 000 PD (95% CI)	Crude IR per 100 000 PD (95% CI)	Standardized IR per 100 000 PD (95% CI)
**Deep vein thrombosis**				
6 mo	0.32 (0.18-0.54)	0.27 (0.14-0.46)	0.23 (0.17-0.31)	0.17 (0.12-0.23)
12 mo	0.25 (0.15-0.40)	0.21 (0.12-0.33)	0.16 (0.12-0.21)	0.12 (0.09-0.16)
**Pulmonary embolism**				
6 mo	1.22 (0.92-1.60)	0.85 (0.62-1.12)	0.66 (0.55-0.79)	0.53 (0.44-0.64)
12 mo	1.46 (1.20-1.76)	0.99 (0.81-1.21)	1.01 (0.90-1.12)	0.79 (0.70-0.88)

Abbreviations: IMID, immune-mediated inflammatory disease; IR, incidence rate; PD, person-days.

The standardized incidence rates of PEs within 6 months were 0.85 (95% CI, 0.62-1.12) per 100 000 PDs among individuals with IMIDs and 0.54 (95% CI, 0.44-0.64) among individuals without IMIDs (Table 3). The standardized incidence rates of PEs within 12 months were 0.99 (95% CI, 0.81-1.21) per 100 000 PDs among individuals with IMIDs and 0.79 (95% CI, 0.70-0.88) among individuals without IMIDs. There was no difference in the risk of PEs among those with and without an IMID (aHR, 1.10; 95% CI, 0.93-1.31).

## Discussion

While IMIDs and COVID-19 are independently associated with VTEs, we observed no additional risk of VTEs among individuals with IMIDs relative to matched individuals without IMIDs following COVID-19 diagnosis after adjusting for COVID-19 vaccination, history of VTE, and comorbidities. However, individuals with IMIDs had a higher risk of VTEs when not adjusting for comorbidities. These findings were consistent in IMID-specific analyses and when separately investigating the risks of DVT and PE.

Both IMIDs and COVID-19 are associated with an increased risk of VTE.^4,5,6,8,9,10,11,12,13,14,15,16,17,18^ Studies examining the additive risk of having an IMID on the post–COVID-19 risk of VTE are sparse. A case-crossover study using health administrative data from a US Veterans Affairs health care system cohort reported that individuals with IBD had higher odds of a VTE following COVID-19 (odds ratio, 8.15; 95% CI, 4.34-15.30) relative to the pre–COVID-19 period.^37^ However, this study did not compare individuals with and without IBD. Another study using Kaiser Permanente data from California also reported elevated risk in patients with IBD and COVID-19 (aHR, 2.43 [95% CI, 1.02-5.80] overall; aHR, 3.46 [95% CI, 1.31-9.13] within 30 days of COVID-19 diagnosis; aHR, 1.05 [95% CI, 0.13-8.46] >30 days from COVID-19 diagnosis).^38^ The wide confidence intervals may indicate imprecision due to smaller sample size of patients with IBD (n = 2006). In addition, this study did not control for vaccination status. Reassuringly, the overall risk of VTEs after COVID-19 in this study (0.26 per 100 person-years [PYs]) was similar to that of the current study (2.18 per 100 000 PDs = 0.8 per 100 PYs).^38^ In a study from the United Kingdom, the risk of thrombosis was similar among those with autoimmune conditions and propensity-matched controls who were hospitalized for COVID-19.^39^ Findings were similar when restricting the analysis to individuals with rheumatologic conditions. In addition, VTE incidence in patients with COVID-19 in the current study was higher than prepandemic population-based VTE rates in Canada (1.29 [95% CI, 1.06-1.53] per 1000 PYs).^40^

The association between IMIDs and VTE following COVID-19 was substantially modified when controlling for comorbidities in the regression analysis. This suggested that comorbidities confounded the association between IMIDs and VTE in the presence of COVID-19. This is a noteworthy finding for clinicians caring for individuals with IMIDs with COVID-19. When treating patients with an IMID and COVID-19, physicians should consider both the underlying COVID-19 severity, individual risk factors, and the presence of comorbidities in determining the need for VTE prophylaxis. In the absence of severe IMID-related inflammation, VTE risk factors, and other comorbid conditions, the IMID itself should not determine the need for VTE prophylaxis.

Our study is strengthened by the use of health administrative data from Canada’s most populous province, including an ethnically diverse population from a large geographic region. Since data include information for all residents eligible for provincial health care coverage (>99%), our study is representative of the Ontario population.

### Limitations

Our study has some limitations. As with all studies using health administrative data, it is subject to misclassification bias. However, we minimized this risk by using validated algorithms for most of the included IMIDs. Our study preceded the Omicron era, and therefore repeated SARS-CoV-2 infections were less common.^41^ Reassuringly, the results of a sensitivity analysis censoring individuals at the time of a second infection were nearly identical to our primary analysis. We also relied on PCR testing for SARS-CoV-2 to identify patients with COVID-19. We may have missed asymptomatic or mild cases, especially during times when the need for testing and/or contact tracing exceeded demand. While all Ontario residents had universal access to PCR testing at no expense, we could not assess whether patients with IMIDs were more likely to be tested than patients without IMIDs. However, all patients admitted to the hospital were routinely tested with PCR. The structure of our data does not allow us to differentiate among individuals admitted to the hospital because of COVID-19, individuals admitted for other reasons but had an incidental positive SARS-CoV-2 test result, or individuals admitted later due to longer-term complications following infection. We therefore could not account for COVID-19 severity. Two-thirds of individuals included in our study had not received 2 or more doses of a COVID-19 vaccine at the time of their infection; a substantial proportion of the individuals included in our study tested positive before vaccines were widely available. We removed IMID-related codes from the CCI to avoid inflation of the CCI in the IMID cohort. In addition, we removed people with cancer in the preceding 5 years from the cohort due to increased risk of VTE. Many other diseases that form parts of the CCI may increase the risk of VTE (eg, myocardial infarction, hemiplegia, peripheral vascular disease). However, removing patients from the cohort with these illnesses would have severely restricted the sample size. In addition, there is no reason to believe that these act as confounders, increasing the VTE risk more in patients with IMIDs than in patients without IMIDs. Our study surprisingly found that the incidence of PE was higher than that of DVT. This may be due to differences in accuracy of the codes/algorithms used to identify these subgroups of VTE. Alternatively, patients with PEs were likely hospitalized more frequently than those with DVTs, so we may have underestimated the risk of DVTs. Our study may also be at risk of residual confounding. For example, Canadian hospitalization data do not include medications. Use of anticoagulants in patients with COVID-19 and/or corticosteroid use could be unmeasured confounders that increase the risk of both severe COVID-19^42,43^ and VTEs^9,44^ in patients with IMIDs. Administrative data do not include clinical characteristics, such as whether there was active inflammation in patients with IMIDs, which may have increased the risk of VTEs.

## Conclusions

In this matched cohort study, we found that the absolute risk of VTE was low in individuals with IMIDs, and the risk was not statistically significantly different from that of the general population after adjusting for comorbidities and vaccination status. Therefore, the presence of an IMID alone should not dictate the use of VTE prophylaxis in patients hospitalized with COVID-19.

## Notes

Supplement 1. **eTable 1.** Algorithms used to identify individuals with immune-mediated inflammatory diseases **eTable 2.** International Classification of Diseases (ICD)-10 codes used to identify individuals developing a venous thromboembolism **eTable 3.** International Classification of Disease (ICD)-10 codes for immune-mediated inflammatory diseases that were excluded from the adapted version of the Charlson Comorbidity Index used in this study **eTable 4.** Previously validated algorithms used to identify individuals with selected comorbidities **eTable 5.** Demographics and clinical characteristics of individuals with immune-mediated inflammatory diseases (IMIDs) and matched people without an IMID on the date of positive COVID-19 test, stratified by type of IMID **eFigure 1.** Forest plot depicting the unadjusted hazard ratio of venous thromboembolism among individuals with and without (reference) an immune-mediated inflammatory disease (IMID) following COVID-19 infection, stratified by type of IMID **eFigure 2.** Forest plot depicting the hazard ratio of venous thromboembolism among individuals with and without (reference) an immune-mediated inflammatory disease (IMID) following COVID-19 infection, adjusted for previous VTE and COVID-19 vaccination stratified by type of IMID
eReferences.

Supplement 2.
Data Sharing Statement
