# Supplementary material for: Venous Thromboembolism After COVID-19 Infection Among People With and Without Immune-Mediated Inflammatory Diseases
Source: JAMA Netw Open. 2023 Oct 9;6(10):e2337020. doi: 10.1001/jamanetworkopen.2023.37020 (PMC10562941; doi:10.1001/jamanetworkopen.2023.37020)
Supplement: Supplement 1. — eTable 1. Algorithms used to identify individuals with immune-mediated inflammatory diseases eTable 2. International Classification of Diseases (ICD)-10 codes used to identify individuals developing a venous thromboembolism eTable 3. International Classification of Disease (ICD)-10 codes for immune-mediated inflammatory diseases that were excluded from the adapted version of the Charlson Comorbidity Index used in this study eTable 4. Previously validated algorithms used to identify individuals with selected comorbidities eTable 5. Demographics and clinical characteristics of individuals with immune-mediated inflammatory diseases (IMIDs) and matched people without an IMID on the date of positive COVID-19 test, stratified by type of IMID eFigure 1. Forest plot depicting the unadjusted hazard ratio of venous thromboembolism among individuals with and without (reference) an immune-mediated inflammatory disease (IMID) following COVID-19 infection, stratified by type of IMID eFigure 2. Forest plot depicting the hazard ratio of venous thromboembolism among individuals with and without (reference) an immune-mediated inflammatory disease (IMID) following COVID-19 infection, adjusted for previous VTE and COVID-19 vaccination stratified by type of IMID eReferences. [file jamanetwopen-e2337020-s001.pdf]

Supplemental Online Content

Khan R, Kuenzig ME, Tang F, et al. Venous thromboembolism after COVID-19 infection among people with and without immune-mediated inflammatory diseases. *JAMA Netw Open.* 2023;6(10):e2337020. doi:10.1001/jamanetworkopen.2023.37020

eTable 1. Algorithms used to identify individuals with immune-mediated inflammatory diseases

eTable 2. International Classification of Diseases (ICD)-10 codes used to identify individuals developing a venous thromboembolism

eTable 3. International Classification of Disease (ICD)-10 codes for immune-mediated inflammatory diseases that were excluded from the adapted version of the Charlson Comorbidity Index used in this study

eTable 4. Previously validated algorithms used to identify individuals with selected comorbidities

eTable 5. Demographics and clinical characteristics of individuals with immune-mediated inflammatory diseases (IMIDs) and matched people without an IMID on the date of positive COVID-19 test, stratified by type of IMID

eFigure 1. Forest plot depicting the unadjusted hazard ratio of venous thromboembolism among individuals with and without (reference) an immune-mediated inflammatory disease (IMID) following COVID-19 infection, stratified by type of IMID

eFigure 2. Forest plot depicting the hazard ratio of venous thromboembolism among individuals with and without (reference) an immune-mediated inflammatory disease (IMID) following COVID-19 infection, adjusted for previous VTE and COVID-19 vaccination stratified by type of IMID

eReferences.

This supplemental material has been provided by the authors to give readers additional information about their work.

© 2023 Khan R et al. *JAMA Network Open.*

eTable 1. Algorithms used to identify individuals with immune-mediated inflammatory diseases

Type of IMID	Diagnostic Codes[a]	Algorithm	Accuracy	Defining IMID Duration
Ankylosing spondylitis	*OHIP:* 720<br>*ICD-9:* 720.0<br>*ICD-10:* M45	• ≥3 physician claims including ≥1 by a rheumatologist or general internist within 3 years, or<br>• ≥1 hospitalization with any of the specified ICD-9 or ICD-10 codes listed as reason for hospitalization or co-morbid condition (primary, secondary, or type 3 diagnosis)	N/A (not validated)	Time from first diagnostic code to date of infection
Inflammatory bowel disease, including subtypes Crohn's disease and ulcerative colitis (1, 2)	*OHIP:* 555, 556<br>*ICD-9:* 555.x, 556.x<br>*ICD-10:* K50.x, K51.x	**Identifying IBD Cases**<br>Pediatrics (<18y):<br>• Underwent colonoscopy: ≥4 physician claims or ≥2 hospitalizations within 3 years, or<br>• Did not undergo colonoscopy: ≥7 physician claims or ≥3 hospitalizations within 3 years<br><br>Adults (18-64y):<br>• ≥5 physician claims or hospitalizations within 4 years<br><br>Seniors (≥65y):<br>• ≥5 physician claims or hospitalizations within 4 years, and<br>• ≥1 prescription for an IBD-related medication	**Identifying IBD Cases[b]**<br>Pediatrics (<18y):<br>• Sens: 89.6% (91.1%)<br>• Spec: >99.9% (99.5%)<br>• PPV: 59.2%<br>• NPV: >99.9%<br><br>Adults (18-64y):<br>• Sens: 76.8% (92.3%)<br>• Spec: 96.2% (99.1%)<br>• PPV: 81.4%<br>• NPV: 95.0%	For incident cases (3y washout period for pediatrics, 8 y washout for adults, seniors):<br>• Time from first diagnostic code to date of infection<br><br>For prevalent cases (did not meet minimum washout period for incident cases): ≥5 years

© 2023 Khan R et al. *JAMA Network Open.*

Type of IMID	Diagnostic Codes[a]	Algorithm	Accuracy	Defining IMID Duration
		**Differentiating subtypes of IBD:**<br>Pediatrics:<br>• 5 of last 7 outpatient contacts<br><br>Adults/Seniors:<br>• 5 of last 9 outpatient contacts	Seniors (≥65y):<br>• Sens: 59.3% (78.3%)<br>• Spec: 99.0% (98.2%)<br>• PPV: 71.1%<br>• NPV: 98.3%<br><br>**Differentiating subtypes of IBD:**<br>Pediatrics:<br>• Sens: 95.1%<br>• Spec: 86.0%<br>• PPV: 92.0%<br>• NPV: 91.2%<br><br>Adults/Seniors:<br>• Accuracy: 91.1%	
Multiple sclerosis (3)	*OHIP:* 340<br>*ICD-9:* 340<br>*ICD-10:* G35	• ≥ 5 physician claims within 2 years, or<br>• ≥1 hospitalization with any of the specified ICD-9 or ICD-10 codes listed as reason for hospitalization or co-morbid condition (primary, secondary, or type 3 diagnosis)	• Sens: 84.2%<br>• Spec: 100%<br>• PPV: 86.0%<br>• NPV: 99.9%	Time from first diagnostic code to date of infection
Polymyalgia rheumatica	*OHIP:* 725<br>*ICD-9:* 725<br>*ICD-10:* M35.3	• ≥3 physician claims including ≥1 with a rheumatologist or	N/A (not validated)	Time from first diagnostic code to date of infection

© 2023 Khan R et al. *JAMA Network Open.*

Type of IMID	Diagnostic Codes[a]	Algorithm	Accuracy	Defining IMID Duration
		general internist within 3 years, or<br>• ≥1 hospitalization with any of the specified ICD-9 or ICD-10 codes listed as reason for hospitalization or co-morbid condition (primary, secondary, or type 3 diagnosis)		
Psoriasis (4)	*OHIP:* 696<br>*ICD-9:* 696.1, 696.8<br>*ICD-10:* L40.0, L40.1, L40.2, L40.3, L40.4, L40.8, L40.9	• ≥3 physician claims, or<br>• ≥1 hospitalization with any of the specified ICD-9 or ICD-10 codes listed as reason for hospitalization or co-morbid condition (primary, secondary, or type 3 diagnosis)	Definite psoriasis:<br>• Spec: 40.6%<br>• PPV: 99.4%<br>• NPV: 71.0%<br><br>Definite and possible psoriasis:<br>• Sens: 98.0%<br>• Spec: 30.8 %<br>• PPV: 99.5%<br>• NPV: 74.7%	Time from first diagnostic code to date of infection
Psoriatic arthritis (4)	*OHIP:* 696 (psoriasis), 721 (spondylarthritis)<br>*ICD-9:* 696.0<br>*ICD-10:* L40.5, M07.0, M07.1, M07.2, M07.3, M09.0	• ≥3 physician claims with a diagnostic code for spondylarthritis including ≥1 by a rheumatologist or general internist and ≥1 physician claim with a diagnostic code for psoriasis (by any specialty), or<br>• ≥1 hospitalization with any of the specified ICD-9 or ICD-10 codes listed as reason for	Definite psoriatic arthritis:<br>• Sens: 48.1%<br>• Spec: 99.9%<br>• PPV: 66.1%<br>• NPV: 99.9%<br><br>Definite and possible psoriatic arthritis:	Time from first diagnostic code to date of infection

© 2023 Khan R et al. *JAMA Network Open.*

Type of IMID	Diagnostic Codes[a]	Algorithm	Accuracy	Defining IMID Duration
		hospitalization or co-morbid condition (primary, secondary, or type 3 diagnosis)	• Sens: 28.6%<br>• Spec: 100%<br>• PPV: 72.7%<br>• NPV: 99.7%	
Rheumatoid arthritis (5, 6)	*OHIP:* 714<br>*ICD-9:* 714<br>*ICD-10:* M05, M06	≥15 years of age and<br>• ≥1 hospitalization, or<br>• ≥3 physician claims including ≥1 with a musculoskeletal specialist (rheumatologist, orthopedic surgeon, or Internal medicine specialist) in two years	Data from selected rheumatology clinics:<br>• Sens: 97%<br>• Spec: 85%<br>• PPV: 76%<br>• NPV:98%<br><br>EMR data:<br>• Sens: 78%<br>• Spec: 100%<br>• PPV: 78%<br>• NPV: 100%	Time from first diagnostic code to date of infection
Systemic autoimmune rheumatic disease	*OHIP:* 710<br>*ICD-9:* 710.0-710.9<br>*ICD-10:* M32, M33, M34, M35.0	• ≥3 physician claims including ≥1 by a rheumatologist or general internist within 3 years, or<br>• ≥1 hospitalization with any of the specified ICD-9 or ICD-10 codes listed as reason for hospitalization or co-morbid condition (primary, secondary, or type 3 diagnosis)	N/A (not validated)	Time from first diagnostic code to date of infection

© 2023 Khan R et al. *JAMA Network Open.*

Type of IMID	Diagnostic Codes[a]	Algorithm	Accuracy	Defining IMID Duration
Uveitis	*OHIP:* 364<br>*ICD-9:* 364.0<br>*ICD-10:* H20.0	• ≥5 physician claims including ≥1 by an ophthalmologist, or<br>• ≥1 hospitalization with any of the specified ICD-9 or ICD-10 codes listed as reason for hospitalization or co-morbid condition (primary, secondary, or type 3 diagnosis)	N/A (not validated)	Time from first diagnostic code to date of infection
Vasculitis	*OHIP:* 446, 447<br>*ICD-9:* 446.0, 446.2, 446.4, 446.5, 446.7<br>*ICD-10:* M30, M31.0, M31.3, M31.4, M31.5, M31.7	• ≥3 physician claims including ≥1 by a rheumatologist, or<br>• ≥1 hospitalization with any of the specified ICD-9 or ICD-10 codes listed as reason for hospitalization or co-morbid condition (primary, secondary, or type 3 diagnosis)	N/A (not validated)	Time from first diagnostic code to date of infection

[a]OHIP codes are an abbreviated list of ICD-9 codes used for outpatient physician billing; ICD-9 codes are included hospital records prior to April 1, 2002; ICD-10 codes are used for hospital records beginning on April 1, 2002
[b]Accuracy of algorithm in the validation cohort is reported in parentheses.
Abbreviations: EMR, electronic medical record; ICD, International Classification of Diseases; NPV, negative predictive value; OHIP, Ontario Health Insurance Plan; PPV, positive predictive value; Sens, sensitivity; Spec, specificity; y, years

© 2023 Khan R et al. *JAMA Network Open.*

eTable 2. International Classification of Diseases (ICD)-10 codes used to identify individuals developing a venous thromboembolism

Type of Venous Thromboembolism	ICD-10 Code	Description
Deep vein thrombosis	I82.2	Embolism and thrombosis of vena cava
	I82.3	Embolism and thrombosis of renal vein
	I82.8	Embolism and thrombosis of other specified veins
	I82.9	Embolism and thrombosis of unspecified vein
	O22.3	Deep phlebothrombosis in pregnancy
	O22.9	Venous complication in pregnancy, unspecified
	O87.1	Deep phlebothrombosis in the puerperium
Pulmonary embolism	I26.x	Pulmonary embolism
Portal vein thrombosis	I81	Portal vein thrombosis
Mesenteric thrombosis	K55.0	Acute vascular disorders of the intestine
Supraventricular vein thrombosis	G08.x	Intracranial and intraspinal phlebitis and thrombophlebitis
	I67.6	Non-pyogenic thrombosis of intracranial venous system

Abbreviations: ICD, International Classification of Diseases

© 2023 Khan R et al. *JAMA Network Open.*

eTable 3. International Classification of Disease (ICD)-10 codes for immune-mediated inflammatory diseases that were excluded from the adapted version of the Charlson Comorbidity Index used in this study

Type of IMID	ICD-10 Codes Excluded
Rheumatoid arthritis	M05, M06
Systemic autoimmune rheumatic disease	M32, M33, M34, M35.0, M35.1, M35.3
Polymyalgia rheumatica	M35.3
Vasculitis	M31.5

Abbreviations: ICD, International Classification of Diseases; IMID, immune-mediated inflammatory diseases

© 2023 Khan R et al. *JAMA Network Open.*

eTable 4. Previously validated algorithms used to identify individuals with selected comorbidities

Comorbidity	Diagnostic Codes[a]	Algorithm	Accuracy
Chronic obstructive pulmonary disease (7)	*OHIP:* 491, 492, 496<br>*ICD-9:* 491, 492, 496<br>*ICD-10:* J41, J42, J43, J44	≥35 years of age and<br>• ≥1 hospitalization or encounter in the CIHI Same Day Surgery database, or<br>• ≥3 physician claims within 2 years	• Sens: 57.5%<br>• Spec: 95.4%<br>• PPV: 81.3%<br>• NPV: 86.7%
Congestive heart failure (8)	*OHIP:*<br>• Diagnostic code: 428<br>• Fee code: Q050<br>*ICD-9:* 428<br>*ICD-10:* I50,0, I50.1, I50.9	≥40 years of age and<br>• ≥1 hospitalization, or<br>• ≥1 physician claim (diagnostic or fee code) or emergency department visit and ≥1 additional encounter of any form (outpatient, emergency department, or hospitalization) within 1 year	• Sens: 84.8%<br>• Spec: 97.0 %<br>• PPV: 55.6%
Diabetes (9, 10)	*OHIP*:<br>• Diagnostic code: 250<br>• Fee codes: Q040, K029, K030, K045, K046<br>*ICD-9:* 250<br>*ICD-10:* E10, E11, E13, E14<br>*Medications:* insulin, oral anti-glycemics	Pediatrics (≤18y):<br>• ≥4 physician claims with diagnostic code for diabetes within 2 years, or<br>• ≥1 physician claims with fee code for diabetes<br>• ≥1 of these encounters occurs before an individual's 19th birthday<br><br>Adults (>18y):<br>• ≥2 physician claims with a diagnostic code for diabetes within 1 year, or<br>• ≥1 prescription for a diabetes medication, or<br>• ≥1 hospitalization<br>• All encounters occur after an individual's 19th birthday	Pediatrics (≤18y):<br>• Sens: 82.8%<br>• Spec: 98.9%<br><br>Adults (>18y):<br>• Sens: 90.0%<br>• Spec: 97.7%

© 2023 Khan R et al. *JAMA Network Open.*

Comorbidity	Diagnostic Codes[a]	Algorithm	Accuracy
		Physician claims, prescription records, or hospitalizations occurring up to 120 days before and 180 days after a hospital record with a diagnostic code for pregnancy care or delivery was considered to be related to gestational diabetes and excluded from the case definitions described above.	• PPV: 82.6%<br>• NPV: 98.8%

Abbreviations: CI, confidence interval; CIHI, Canadian Institute for Health Information; ICD, International Classification of Diseases; NPV, negative predictive value; OHIP, Ontario Health Insurance Plan; PPV, positive predictive value; Sens, sensitivity; Spec, specificity; y, years

[a]OHIP codes are an abbreviated list of ICD-9 codes used for outpatient physician billing; ICD-9 codes are included hospital records prior to April 1, 2002; ICD-10 codes are used for hospital records beginning on April 1, 2002

© 2023 Khan R et al. *JAMA Network Open.*

eTable 5. Demographics and clinical characteristics of individuals with immune-mediated inflammatory diseases (IMIDs) and matched people without an IMID on the date of positive COVID-19 test, stratified by type of IMID

Characteristic	People with Specified IMID	Matched People without an IMID
**Ankylosing Spondylitis**		
n	1453	6600
Age (years), mean (SD)	50.8 (15.7)	49.5 (14.9)
Female, n (%)	699 (48.1)	3151 (48.7)
Rural, n (%)	50 (3.4)	100 (1.5)
Mean neighborhood income quintile, n (%)		
1 (lowest)	301 (20.7)	1378 (20.9)
2	282 (19.4)	1293 (19.6)
3	300 (20.6)	1398 (21.2)
4	293 (20.2)	1313 (19.9)
5 (highest)	277 (19.1)	1218 (18.5)
Previous VTE, n (%)	13 (0.9)	41 (0.6)
Chronic obstructive pulmonary disease, n (%)	58 (4.0)	112 (1.7)
Congestive heart failure, n (%)	52 (3.6)	137 (2.1)
Diabetes, n (%)	268 (18.4)	1066 (16.2)
Charlson Comorbidity Index, median (Q1, Q3)	1 (0, 2)	0 (0, 1)
IMID duration, n (%)		
<1 year	79 (5.4)	N/A
1 to <3 years	189 (13.0)	N/A
3 to <5 years	179 (12.3)	N/A
≥5 years	1006 (69.2)	N/A
Number of additional IMIDs, n (%)		
0	830 (57.1)	N/A
1	413 (28.4)	N/A
≥2	210 (14.5)	N/A
COVID-19 vaccination, n (%)	447 (30.8)	1915 (29.0)
**Inflammatory Bowel Disease**		
n	4331	19,903
Age (years), mean (SD)	46.0 (17.2)	44.7 (16.5)
Female, n (%)	2166 (50.0)	9892 (49.7)
Rural, n (%)	150 (3.5)	328 (1.6)
Mean neighborhood income quintile, n (%)		
1 (lowest)	754 (17.4)	3447(17.3)
2	856 (19.8)	3944 (19.8)
3	900 (20.8)	4124 (20.7)
4	894 (20.6)	4086 (20.5)

© 2023 Khan R et al. *JAMA Network Open.*

Characteristic	People with Specified IMID	Matched People without an IMID
5 (highest)	927 (21.4)	4302 (21.6)
Previous VTE, n (%)	63 (1.5)	96 (0.5)
Chronic obstructive pulmonary disease, n (%)	126 (2.9)	287 (1.4)
Congestive heart failure, n (%)	128 (3.0)	297 (1.5)
Diabetes, n (%)	563 (13.0)	2521 (12.7)
Charlson Comorbidity Index, median (Q1, Q3)	0 (0, 1)	0 (0, 1)
Type of IBD, n (%)		
Crohn's disease	1888 (43.6)	N/A
Ulcerative colitis	2260 (52.2)	N/A
IBD-U	183 (4.2)	N/A
IMID duration, n (%)		
<1 year	68 (1.6)	N/A
1 to <3 years	340 (7.9)	N/A
3 to <5 years	407 (9.4)	N/A
≥5 years	3516 (81.2)	N/A
Number of additional IMIDs, n (%)		
0	3807 (87.9)	N/A
1	423 (9.8)	N/A
≥2	101 (2.3)	N/A
COVID-19 vaccination, n (%)	1469 (33.9)	6521 (32.8)
**Multiple Sclerosis**		
n	1954	8799
Age (years), mean (SD)	50.6 (15.6)	49.3 (15.1)
Female, n (%)	1381 (70.7)	6201 (70.6)
Rural, n (%)	73 (3.7)	159 (1.8)
Mean neighborhood income quintile, n (%)		
1 (lowest)	440 (22.5)	1989 (22.7)
2	413 (21.1)	1847 (21.0)
3	376 (19.2)	1718 (19.6)
4	381 (19.5)	1707 (19.4)
5 (highest)	344 (17.6)	1518 (17.3)
Previous VTE, n (%)	40 (2.0)	43 (0.5)
Chronic obstructive pulmonary disease, n (%)	59 (3.0)	184 (2.1)
Congestive heart failure, n (%)	84 (4.3)	189 (2.2)
Diabetes, n (%)	306 (15.7)	1304 (14.9)
Charlson Comorbidity Index, median (Q1, Q3)	1 (0, 2)	0 (0, 1)
IMID duration, n (%)		
<1 year	103 (5.3)	N/A

© 2023 Khan R et al. *JAMA Network Open.*

Characteristic	People with Specified IMID	Matched People without an IMID
1 to <3 years	229 (11.7)	N/A
3 to <5 years	162 (8.3)	N/A
≥5 years	1,460 (74.7)	N/A
Number of additional IMIDs, n (%)		
0	1805 (92.4)	N/A
1	126 (6.5)	N/A
≥2	23 (1.2)	N/A
COVID-19 vaccination, n (%)	522 (26.7)	2425 (27.6)
**Polymyalgia Rheumatica**		
n	535	1902
Age (years), mean (SD)	76.6 (13.1)	74.5 (13.6)
Female, n (%)	354 (66.2)	1241 (65.2)
Rural, n (%)	12 (2.2)	23 (1.2)
Mean neighborhood income quintile, n (%)		
1 (lowest)	116 (21.7)	449 (23.6)
2	120 (22.4)	436 (22.9)
3	111 (20.7)	391 (20.6)
4	98 (18.3)	356 (18.7)
5 (highest)	90 (16.8)	270 (14.2)
Previous VTE, n (%)	17 (3.2)	30 (1.6)
Chronic obstructive pulmonary disease, n (%)	60 (11.2)	155 (8.1)
Congestive heart failure, n (%)	114 (21.3)	293 (15.4)
Diabetes, n (%)	212 (39.6)	722 (38.0)
Charlson Comorbidity Index, median (Q1, Q3)	2 (1, 5)	2 (1, 3)
IMID duration, n (%)		
<1 year	60 (11.2)	N/A
1 to <3 years	111 (20.7)	N/A
3 to <5 years	88 (16.4)	N/A
≥5 years	276 (51.6)	N/A
Number of additional IMIDs, n (%)		
0	359 (67.1)	N/A
1	139 (26.0)	N/A
≥2	37 (6.9)	N/A
COVID-19 vaccination, n (%)	97 (18.1)	321 (16.9)
**Psoriatic Arthritis**		
N	1134	5085
Age (years), mean (SD)	54.1 (14.6)	52.7 (14.0)
Female, n (%)	584 (51.5)	2553 (50.2)
Rural, n (%)	35 (3.1)	95 (1.9)

© 2023 Khan R et al. *JAMA Network Open.*

Characteristic	People with Specified IMID	Matched People without an IMID
Mean neighborhood income quintile, n (%)		
1 (lowest)	252 (22.2)	1116 (21.9)
2	209 (18.4)	961 (18.9)
3	210 (18.5)	971 (19.1)
4	231 (20.4)	1027 (20.2)
5 (highest)	232 (20.5)	1010 (19.9)
Previous VTE event, n (%)	15 (1.3)	36 (0.7)
Chronic obstructive pulmonary disease, n (%)	40 (3.5)	123 (2.4)
Congestive heart failure, n (%)	56 (4.9)	128 (2.5)
Diabetes, n (%)	292 (25.7)	901 (17.7)
Charlson Comorbidity Index, median (Q1, Q3)	1 (0, 2)	0 (0, 1)
IMID duration, n (%)		
<1 year	26 (2.3)	N/A
1 to <3 years	64 (5.6)	N/A
3 to <5 years	81 (7.1)	N/A
≥5 years	963 (84.9)	N/A
Number of additional IMIDs, n (%)		
0	162 (14.3)	N/A
1	622 (54.9)	N/A
≥2	350 (30.9)	N/A
COVID-19 vaccination, n (%)	355 (31.3)	1478 (29.1)
**Psoriasis**		
n	10,739	48,743
Age (years), mean (SD)	49.9 (18.2)	48.3 (17.4)
Female, n (%)	5424 (50.5)	24,500 (50.3)
Rural, n (%)	342 (3.2)	763 (1.6)
Mean neighborhood income quintile, n (%)		
1 (lowest)	2252 (21.0)	10,273 (21.1)
2	2147 (20.0)	9772 (20.0)
3	2199 (20.5)	10,103 (20.7)
4	2126 (19.8)	9631 (19.8)
5 (highest)	2015 (18.8)	8964 (18.4)
Previous VTE, n (%)	97 (0.9)	322 (0.7)
Chronic obstructive pulmonary disease, n (%)	390 (3.6)	1034 (2.1)
Congestive heart failure, n (%)	448 (4.2)	1284 (2.6)
Diabetes, n (%)	2138 (19.9)	7719 (15.8)
Charlson Comorbidity Index, median (Q1, Q3)	0 (0, 2)	0 (0, 1)

© 2023 Khan R et al. *JAMA Network Open.*

Characteristic	People with Specified IMID	Matched People without an IMID
IMID duration, n (%)		
<1 year	299 (2.8)	N/A
1 to <3 years	782 (7.3)	N/A
3 to <5 years	783 (7.3)	N/A
≥5 years	8875 (82.6)	N/A
Number of additional IMIDs, n (%)		
0	9302 (86.6)	N/A
1	1051 (9.8)	N/A
≥2	386 (3.6)	N/A
COVID-19 vaccination, n (%)	2996 (27.9)	13,649 (28.0)
**Rheumatoid Arthritis**		
n	5883	24,993
Age (years), mean (SD)	59.5 (17.4)	57.2 (16.9)
Female, n (%)	4329 (73.6)	18,352 (73.4)
Rural, n (%)	206 (3.5)	393 (1.6)
Mean neighborhood income quintile, n (%)		
1 (lowest)	1340 (22.8)	5721 (22.9)
2	1274 (21.7)	5446 (21.8)
3	1267 (21.5)	5524 (22.1)
4	1061 (18.0)	4490 (18.0)
5 (highest)	941 (16.0)	3812 (15.3)
Previous VTE, n (%)	125 (2.1)	211 (0.8)
Chronic obstructive pulmonary disease, n (%)	440 (7.5)	869 (3.5)
Congestive heart failure, n (%)	483 (8.2)	1175 (4.7)
Diabetes, n (%)	1579 (26.8)	5777 (23.1)
Charlson Comorbidity Index, median (Q1, Q3)	2 (1, 3)	0 (0, 2)
IMID duration, n (%)		
<1 year	271 (4.6)	N/A
1 to <3 years	797 (13.5)	N/A
3 to <5 years	790 (13.4)	N/A
≥5 years	4,025 (68.4)	N/A
Number of additional IMIDs, n (%)		
0	4480 (76.2)	N/A
1	998 (17.0)	N/A
≥2	405 (6.9)	N/A
COVID-19 vaccination, n (%)	1357 (23.1)	5669 (22.7)
**Systemic Autoimmune Rheumatic Disease**		
n	2506	11,265
Age (years), mean (SD)	51.7 (17.5)	50.0 (16.6)

© 2023 Khan R et al. *JAMA Network Open.*

Characteristic	People with Specified IMID	Matched People without an IMID
Female, n (%)	2141 (85.4)	9674 (85.9)
Rural, n (%)	78 (3.1)	173 (1.5)
Mean neighborhood income quintile, n (%)		
1 (lowest)	563 (22.5)	2566 (22.8)
2	501 (20.0)	2234 (19.8)
3	534 (21.3)	2412 (21.4)
4	451 (18.0)	2056 (18.3)
5 (highest)	457 (18.2)	1997 (17.7)
Previous VTE, n (%)	86 (3.4)	86 (0.8)
Chronic obstructive pulmonary disease, n 9%)	127 (5.1)	218 (1.9)
Congestive heart failure, n (%)	174 (6.9)	283 (2.5)
Diabetes, n (%)	480 (19.2)	1827 (16.2)
Charlson Comorbidity Index, median (Q1, Q3)	1 (0, 3)	0 (0, 1)
IMID duration, n (%)		
<1 year	176 (7.0)	N/A
1 to <3 years	373 (14.9)	N/A
3 to <5 years	294 (11.7)	N/A
≥5 years	1663 (66.4)	N/A
Number of additional IMIDs, n (%)		
0	1854 (74.0)	N/A
1	555 (22.2)	N/A
≥2	97 (3.9)	N/A
COVID-19 vaccination, n (%)	664 (26.5)	3025 (26.9)
**Uveitis**		
n	3000	12,742
Age (years), mean (SD)	57.8 (19.8)	55.0 (19.0)
Female, n (%)	1635 (54.5)	6805 (53.4)
Rural, n (%)	84 (2.8)	191 (1.5)
Mean neighborhood income quintile, n (%)		
1 (lowest)	739 (24.6)	3144 (24.7)
2	606 (20.2)	2593 (20.4)
3	599 (20.0)	2610 (20.5)
4	550 (18.3)	2330 (18.3)
5 (highest)	506 (16.9)	2065 (16.2)
Previous VTE, n (%)	40 (1.3)	104 (0.8)
Chronic obstructive pulmonary disease, n (%)	176 (5.9)	433 (3.4)
Congestive heart failure, n (%)	226 (7.5)	593 (4.7)
Diabetes, n (%)	856 (28.5)	2692 (21.1)

© 2023 Khan R et al. *JAMA Network Open.*

Characteristic	People with Specified IMID	Matched People without an IMID
Charlson Comorbidity Index, median (Q1, Q3)	1 (0, 2)	0 (0, 2)
IMID duration, n (%)		
<1 year	119 (4.0)	N/A
1 to <3 years	195 (6.5)	N/A
3 to <5 years	244 (8.1)	N/A
≥5 years	2442 (81.4)	N/A
Number of additional IMIDs, n (%)		
0	2478 (82.6)	N/A
1	420 (14.0)	N/A
≥2	102 (3.4)	N/A
COVID-19 vaccination, n (%)	706 (23.5)	3094 (24.3)

Abbreviations: IBD, inflammatory bowel disease; IBD-U, inflammatory bowel disease type unclassifiable; IMID, immune-mediated inflammatory disease; VTE, venous thromboembolism

© 2023 Khan R et al. *JAMA Network Open.*

eFigure 1. Forest plot depicting the unadjusted hazard ratio of venous thromboembolism among individuals with and without (reference) an immune-mediated inflammatory disease (IMID) following COVID-19 infection, stratified by type of IMID

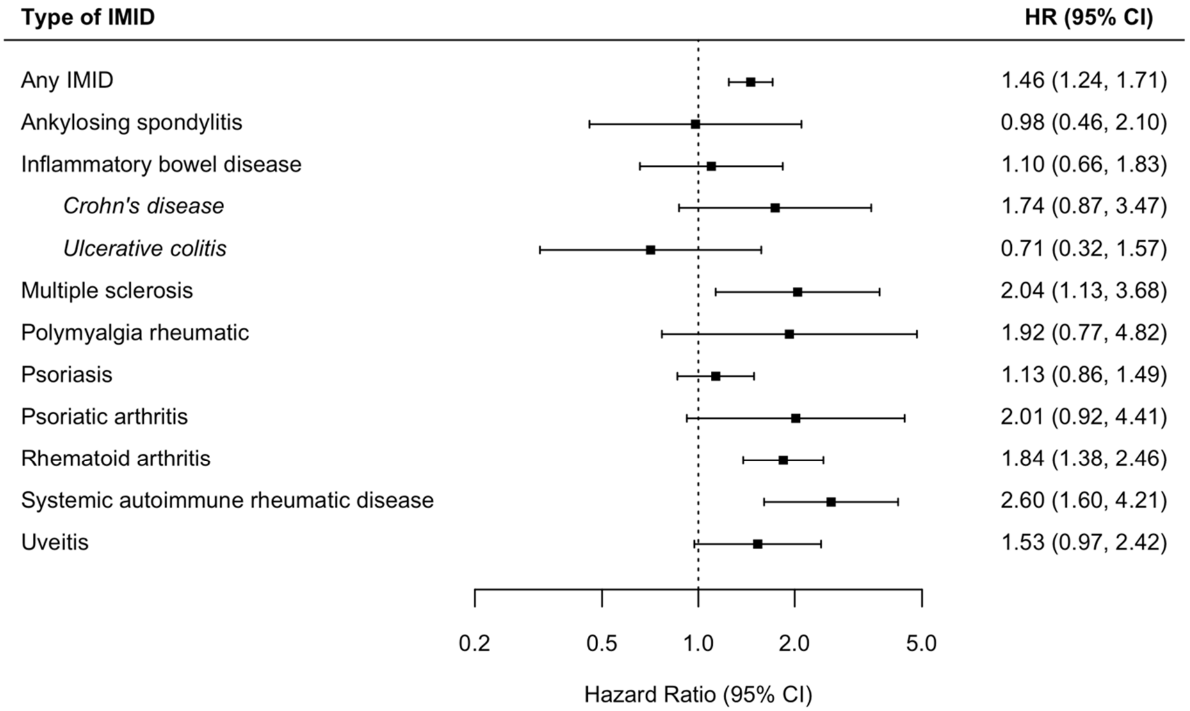

© 2023 Khan R et al. *JAMA Network Open.*

eFigure 2. Forest plot depicting the hazard ratio of venous thromboembolism among individuals with and without (reference) an immune-mediated inflammatory disease (IMID) following COVID-19 infection, adjusted for previous VTE and COVID-19 vaccination stratified by type of IMID

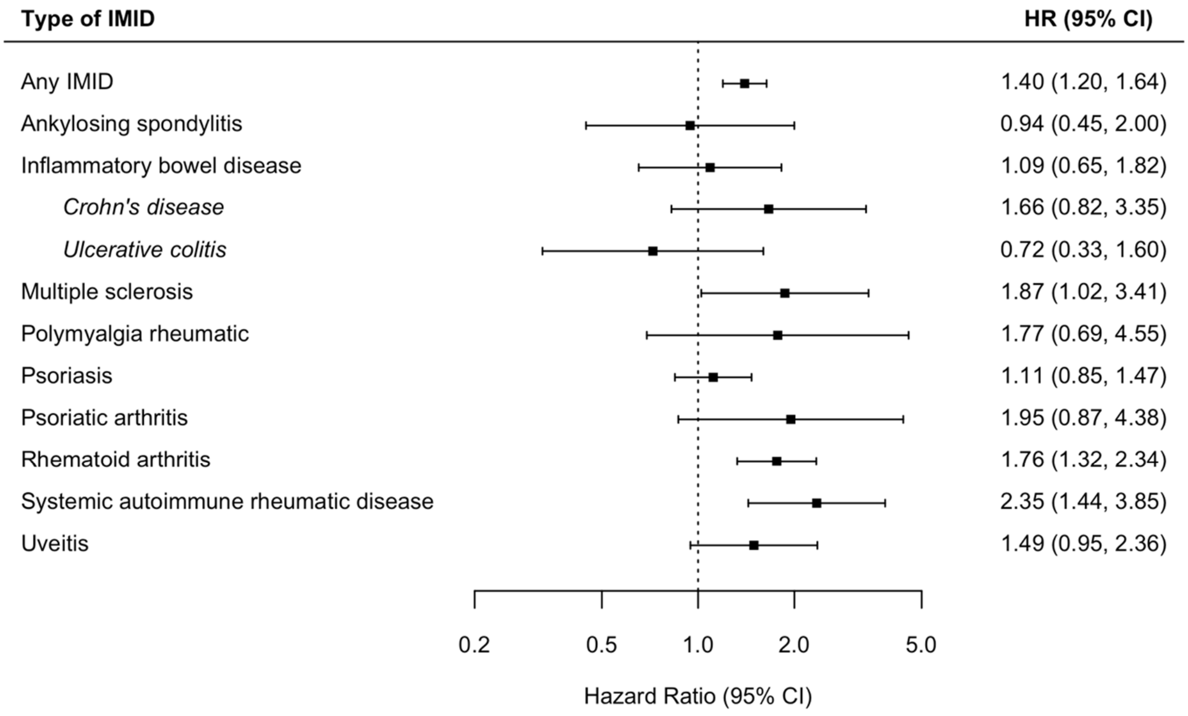

© 2023 Khan R et al. *JAMA Network Open.*

eReferences

1. Benchimol EI, Guttmann A, Griffiths AM, Rabeneck L, Mack DR, Brill H, et al. Increasing incidence of paediatric inflammatory bowel disease in Ontario, Canada: evidence from health administrative data. Gut. 2009;58(11):1490-7.
2. Benchimol EI, Guttmann A, Mack DR, Nguyen GC, Marshall JK, Gregor JC, et al. Validation of international algorithms to identify adults with inflammatory bowel disease in health administrative data from Ontario, Canada. J Clin Epidemiol. 2014;67(8):887-96.
3. Widdifield J, Ivers NM, Young J, Green D, Jaakkimainen L, Butt DA, et al. Development and validation of an administrative data algorithm to estimate the disease burden and epidemiology of multiple sclerosis in Ontario, Canada. Mult Scler. 2015;21(8):1045-54.
4. Eder L, Widdifield J, Rosen CF, Alhusayen R, Cheng SY, Young J, et al. Identifying and Characterizing Psoriasis and Psoriatic Arthritis Patients in Ontario Administrative Data: A Population-based Study From 1991 to 2015. J Rheumatol. 2020;47(11):1644-51.
5. Widdifield J, Bombardier C, Bernatsky S, Paterson JM, Green D, Young J, et al. An administrative data validation study of the accuracy of algorithms for identifying rheumatoid arthritis: the influence of the reference standard on algorithm performance. BMC Musculoskelet Disord. 2014;15:216.
6. Widdifield J, Bernatsky S, Paterson JM, Tu K, Ng R, Thorne JC, et al. Accuracy of Canadian health administrative databases in identifying patients with rheumatoid arthritis: a validation study using the medical records of rheumatologists. Arthritis Care Res (Hoboken). 2013;65(10):1582-91.
7. Gershon AS, Wang C, Guan J, Vasilevska-Ristovska J, Cicutto L, To T. Identifying individuals with physcian diagnosed COPD in health administrative databases. COPD. 2009;6(5):388-94.
8. Schultz SE, Rothwell DM, Chen Z, Tu K. Identifying cases of congestive heart failure from administrative data: a validation study using primary care patient records. Chronic Dis Inj Can. 2013;33(3):160-6.
9. Lipscombe LL, Hwee J, Webster L, Shah BR, Booth GL, Tu K. Identifying diabetes cases from administrative data: a population-based validation study. BMC Health Serv Res. 2018;18(1):316.
10. Guttmann A, Nakhla M, Henderson M, To T, Daneman D, Cauch-Dudek K, et al. Validation of a health administrative data algorithm for assessing the epidemiology of diabetes in Canadian children. Pediatr Diabetes. 2010;11(2):122-8.

© 2023 Khan R et al. *JAMA Network Open.*
